# Blepharophimosis, Ptosis, and Epicanthus Inversus Syndrome: A Simple Remedy for Challenging Cases

**DOI:** 10.7759/cureus.27432

**Published:** 2022-07-29

**Authors:** Saba Alkhairy, Hania Saeed, Samir Saeed

**Affiliations:** 1 Ophthalmology, Dow University of Health Sciences, Dow International Medical College, Karachi, PAK

**Keywords:** ptosis, two stage, z-plasty, genetic mutations, eyelid malformation, telecanthus, epicanthus inversus, blepharophimosis

## Abstract

A 14-year-old male presented to the outpatient department of ophthalmology with complaints of visual impairment. The patient was assessed with a detailed history and physical examination. Marked amblyopia was observed on inspection, and his best-corrected vision was 6/36 in both eyes with no further improvement. Both the anterior and posterior segments of the eyes were normal. A diagnosis of blepharophimosis, ptosis, and epicanthus inversus syndrome (BPES) was suspected. Surgery was initiated in two stages, with the first stage utilizing Mustarde's double Z-plasty to correct the epicanthus inversus and telecanthus. The second stage was done three months later, involving a tarsofrontalis sling with prolene sutures to correct ptosis. The success of this operation speaks to the efficacy of a two-stage procedure for remedying a syndrome as complex as BPES.

## Introduction

Blepharophimosis, ptosis, and epicanthus inversus syndrome (BPES) is a rare congenital and developmental condition that manifests with eyelid malformations. It is mainly characterized by four features at birth: blepharophimosis, ptosis, epicanthus inversus, and telecanthus. Blepharophimosis presents as a shortening of the horizontal orbital fissure, while ptosis is drooping of the eyelids. Epicanthus inversus is an upward fold of skin from the lower eyelid, and telecanthus is an abnormally increased distance between the medial canthus [1].

BPES exhibits autosomal dominant inheritance in most cases, but it may also occur sporadically. Adding to the complexity of this syndrome is that there are two phenotypes of BPES: type I and type II. Type I BPES is associated with eyelid abnormalities and, notably, female infertility due to premature ovarian failure. Type II BPES only includes eyelid abnormalities with no associated systemic features [2]. 

Both types of BPES are caused by mutations in the Forkhead Box L2 (FOXL2) gene, which encodes for a transcription factor that is expressed in the developmental stages of mesenchyme in eyelids and ovaries [3]. Because of this genetic involvement, molecular genetic testing for a pathogenic variant in FOXL2, along with clinical findings that include the four eyelid abnormalities, can be used to establish a diagnosis of BPES [4]. Treatment for BPES usually consists of surgery done in two stages. The first stage involves Mustarde's double Z-plasty with transnasal fixation with 4-0 prolene. The second stage is performed after three months, and this involves a lateral canthoplasty for horizontal lid fissure widening and a tarsofrontalis sling with a silicone rod for ptosis correction [5].

## Case presentation

**Figure 1:**
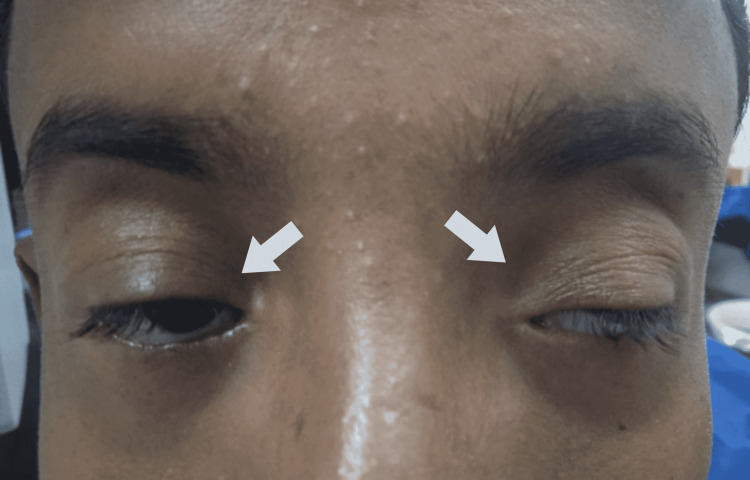
Preoperative image in which ocular features consistent with BPES were observed BPES - blepharophimosis, ptosis, and epicanthus inversus syndrome

A 14-year-old male presented to the eye outpatient department (OPD) of Dow University of Health Sciences (DUHS) in Karachi, Pakistan, with complaints of visual impairment due to the inability to fully open the eye (Figure 1) in September 2020. The patient is from Larkana and was accompanied by his biological father. He denied any other ocular or systemic complaints. However, he did have a visual impairment which was determined to be due to refractive error amblyopia, and his best-corrected vision was 6/36 in both eyes with no further improvement. An inquiry of the patient's family history for this condition revealed a negative finding. The patient has no significant past medical or surgical history and has never consumed betel nuts, drugs, or alcohol. Ocular movements, alignment, fundus, and slit-lamp examinations were normal in the patient. The patient's antenatal, natal, and postnatal histories were also insignificant. 

**Figure 2:**
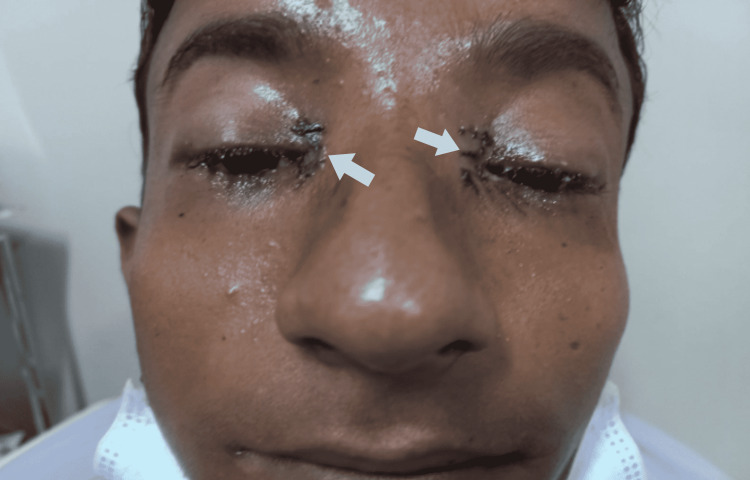
Postoperative image after stage one correction of BPES BPES - blepharophimosis, ptosis, and epicanthus inversus syndrome

**Figure 3:**
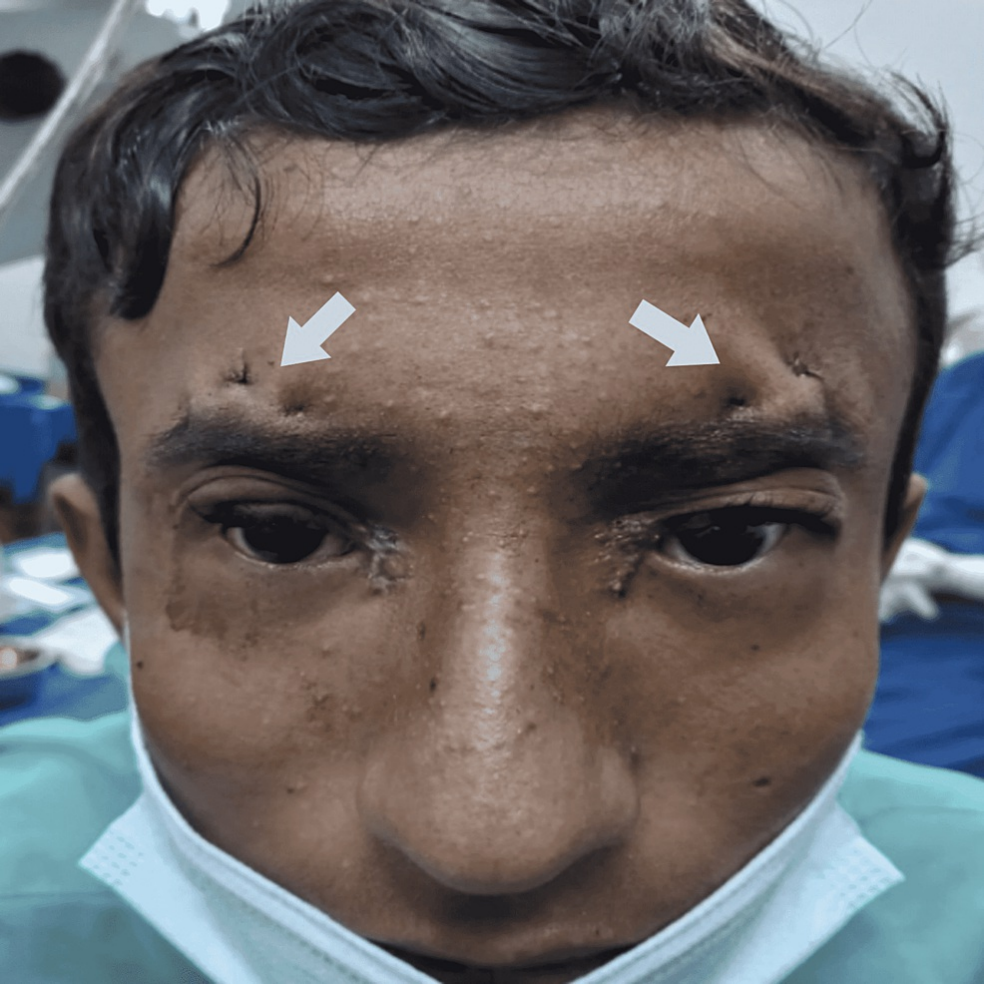
Postoperative image after stage two correction of BPES BPES - blepharophimosis, ptosis, and epicanthus inversus syndrome

The first in what would become a two-part series of operations was performed for the purpose of correcting BPES. The first stage of surgery, Mustarde's double Z-plasty, was used to correct the epicanthic fold and telecanthus (Figure 2). In this procedure, the subcutaneous tissue was detached, and the medial canthal tendon was reduced by using a suture fixation just behind the medial canthal tendon insertion. In the second stage, bilateral ptosis correction was done using a frontalis sling with prolene 4/0 suture three months after Mustarde's double Z-plasty procedure (Figure 3). In this procedure, the elevating function of the frontalis muscle was shifted to the ptotic eyelid by creating a sling between the frontalis muscle and the eyelid, making it possible to suspend the eyelid from the brow. 

## Discussion

BPES is an atypical genetic disorder with an approximate incidence of one in 50,000 births. The cosmetic disfigurement associated with BPES can lead to social and emotional dilemmas that can impact the patient's daily lifestyle [6]. Up to 75% of individuals with BPES have detectable FOXL2 mutation, which leads to haploinsufficiency. A majority of these mutations are intragenic and can be subdivided into indel frameshift, in-frame deletions, nonsense, missense, and duplications [3].

Patients with BPES require a single or multistage correction during childhood. There is considerable controversy regarding the best surgical approach to this condition. The advantage of a one-stage correction is that it requires less surgical time and avoids additional anesthetic hazards. However, one disadvantage to this approach is that medial canthoplasty may be unpredictable. Additionally, ptosis correction generates a vertical force, while Mustarde's double Z-plasty causes a horizontal force. So when both of these procedures are performed simultaneously, as in a one-stage correction, the tension created by each surgery opposes the other, and this can cause loosening of epicanthal correction and poor elevation of the upper eyelid [5]. 

A two-stage approach for BPES can be quite effective. There are several ways to perform a two-stage approach. The first stage includes epicanthus and telecanthus correction, which is usually performed using Mustarde's double Z-plasty or V-Y procedure. The second stage involves ptosis correction. For some patients, lateral canthoplasty is used to extend the palpebral fissure length and can be done simultaneously with medial canthoplasty or after correction of ptosis. Ptosis correction is achieved via frontalis suspension, which itself involves many surgical techniques and the use of sling materials [7].

Another approach is medical canthoplasty, which is performed in the first stage to eliminate the epicanthal fold. This medial canthoplasty can be performed as a Y to V procedure or as Z-epicanthoplasty. For correction of telecanthus, some prefer to use transnasal wiring, but this approach is difficult to implement and has an increased risk of infection. Plication of medial canthal tendon with fixation of subcutaneous tissue to canthal tendon is effective for correcting telecanthus. For ptosis correction, a frontalis suspension is usually preferred because most patients with BPES have minimal levator function [8].

## Conclusions

In conclusion, little recent literature is available on BPES due to the consequence of its rarity. There are various methods described and used for correcting BPES. Our study proposed a two-stage procedure that is the most successful in attenuating the functional and cosmetic deficiencies of this unique and complex syndrome. There was a significant improvement in blepharophimosis, ptosis, epicanthus inversus, and a decreased inner canthal distance after this surgery. Effective and timely management of BPES can prevent amblyopia and significantly improve cosmesis. After this surgical procedure, the patient now has relief from adverse psychosocial consequences. 
